# Applications of the prediction of satisfaction design for monitoring single-arm phase II trials

**DOI:** 10.1371/journal.pone.0305814

**Published:** 2024-09-26

**Authors:** Zohra Djeridi, Ahlem Ghouar, Hamid Boulares, Mohamed Bouye

**Affiliations:** 1 Department of Mathematics, University of Jijel, Jijel, Algeria; 2 ACED Laboratory University of 8 May 1945 Guelma, Guelma, Algeria; 3 Preparatory Department, Higher School of Management Sciences-Annaba, Annaba, Algeria; 4 Department of Mathematics, University of 8 May 1945 Guelma, Guelma, Algeria; 5 Department of Mathematics, College of Science, King Khalid University, Abha, Saudi Arabia; Texas A&M University, UNITED STATES

## Abstract

Prediction of satisfaction design, with binary endpoints, is an innovative strategy for phase II trials. We explain this hybrid frequentist-Bayesian strategy with an adept statistical plan and thorough findings, incorporating a description of study design features such as the sample size and the beta prior distribution, to simplify the Bayesian design. We also provide a set of tables and figures ranging from the stopping boundary for futility to the prediction of satisfaction, performance (type I error, power, and the probability of early termination PET), and sensitivity analysis for prediction of satisfaction. The statistical plan includes the operating characteristics through simulation study. Several trial examples from phase II lung cancer studies demonstrate the approach’s practical use. The prediction of satisfaction design presents a flexible method in clinical study. This statistical study adds value to the application by broadening its scope.

## 1 Introduction

Bayesian analysis of phase II clinical trials has become an integral part of the development and dissemination of therapeutic drugs, surgical and technological interventions [1–3]. The routine design and analysis of the phase II clinical trials is a constant source of inspiration and creativity to Bayesians who are interested in medical statistics. They find a combination of frequentist (classical) theoretic use of type I and type II errors, Fisherian use of tail-areas as standardised measures of evidence, the predictive probability and stopping boundaries for sequential analysis carefully preserving type I error. [2, 4, 5].

Recently many works have been done in a sequential setting, where a trial has a several interim analysis and it can be stopped for futility and / or efficacy after any stage, rather than waiting until the completion of the entire trial. It allows for ongoing monitoring of the accumulating data and enables early termination or modification of the trial based on predefined stopping rules. For example, in phase II cancer clinical trials, multi-stage designs are frequently employed. Especially for phase II single-arm (uncontrolled) design, many Bayesian sequential techniques have been proposed, including posterior probability, predictive probability, and even combining with a frequentist approach [6]. For example, Tall and Simon [7] utilized posterior probability to build stopping criteria for continuously monitoring schema, whereas Lee and Liu [8], Saville et al. [3] and Chen et al [6] used predictive probability to construct the boundary for multi-stage designs. Sambuchini, in 2021, [9], described a number of Bayesian approaches to perform interim analysis in single-arm trials based on a binary response variable. Djeridi and Merabet 2019 [10] proposed a hybrid frequentist Bayesian approach to define the stopping boundary by using the prediction of satisfaction. The efficacy *θ*_0_ of an experimental therapy E is evaluated in this design using data from an uncontrolled (single-arm, *i*.*e*., all participants receive the same treatment or intervention being investigated) trial of E. The experiment will continue until E demonstrates a high prediction of satisfaction to the promising or not promising, or until a pre-set maximum sample size is achieved. The idea is to assess the probability of achieving a desired outcome (that assures the efficacy of the therapy) at the trial’s scheduled end point based on the observed interim result.

The major problem in sequential testing procedures is the inflation of significance level through repeatedly analysing the data as it accumulates. So, the use of the index of satisfaction which is an increasing function of the p-value of the significant result, if the trial will continue to its term, to build the index of satisfaction can resolve this problem to ensure that the type I error of the final analysis is controlled. Furthermore, by using Bayesian predictive techniques, the prediction of satisfaction is simpler to interpret and more helpful for decision making.

Bayesian group sequential approaches, in general, are not intended to optimize frequentist operating characteristics, but rather to offer stopping criteria for eventual decision making [1]. But, the prediction of satisfaction design ensures the overall false positive rate by employing the concept of the p-value of a test concluding a positive result by the conclusion of the trial based on the present stage’s cumulative information. In this study, we claim that the prediction of satisfaction design offers sufficient test power. Also, we discuss sample size selection and monitoring criteria. Numerical implementation directions are presented. Based on the predictive distribution of the observed success rate, this strategy provides criteria for early termination of trials that are unlikely to lead to conclusive results.

The outline of the paper is as follows: in section 2, we establish the concept of index and prediction of satisfaction in a sequential setting. The use of the prior distribution and the maximum sample size are also discussed with some modelling considerations. In section 3, we discuss the sensibility analysis for multi-stage plans. Then, several real examples that illustrate the use of this approach in the design of clinical trials are presented in section 4. Finally, the paper closes with a discussion.

## 2 Analytical method

### 2.1 Concept of the index of satisfaction in a sequential setting

Typically statisticians perform a hypothesis test, utilize the concept of the index of satisfaction to assume discovering a significant conclusion by the end of the study; that is, rejecting the null hypothesis *H*_0_ of an ineffective treatment.

Correlatively, this statistician is especially more satisfied when, based on the experimental data this effect appears more significant. It is more interesting to consider the level at which the result always seems significant. Therefore, if no significant impact is observed, the satisfaction indexes are null, and in the opposite scenario, they are an increasing function of the conventional indicator of significance, which is the p-value of the final result in the test theory [11]. If a trial yields a positive result, early termination allows the new product to be used sooner; if a bad result is yielded, early termination guarantees that resources are not lost [12]. So, it is more useful to predict the level of satisfaction by a weighted average of this index with regard to the predictive probabilities over all the points, if observed, would just lead to the rejection of the null hypothesis, which is conditioned by the first stage outcome [13]. Given the number of current status, the predicted level of satisfaction is the chance of achieving treatment effectiveness at the conclusion of the trial.

This approach can be beneficial for interim monitoring, according the prediction of satisfaction that surpasses a pre-specified threshold to indicate the possibility of early termination. If the prediction of satisfaction is lower than the threshold, the therapy is regarded ineffective, and the action of early termination is contemplated. When it is close to one, the likelihood of success is high.

**Fig 1:**
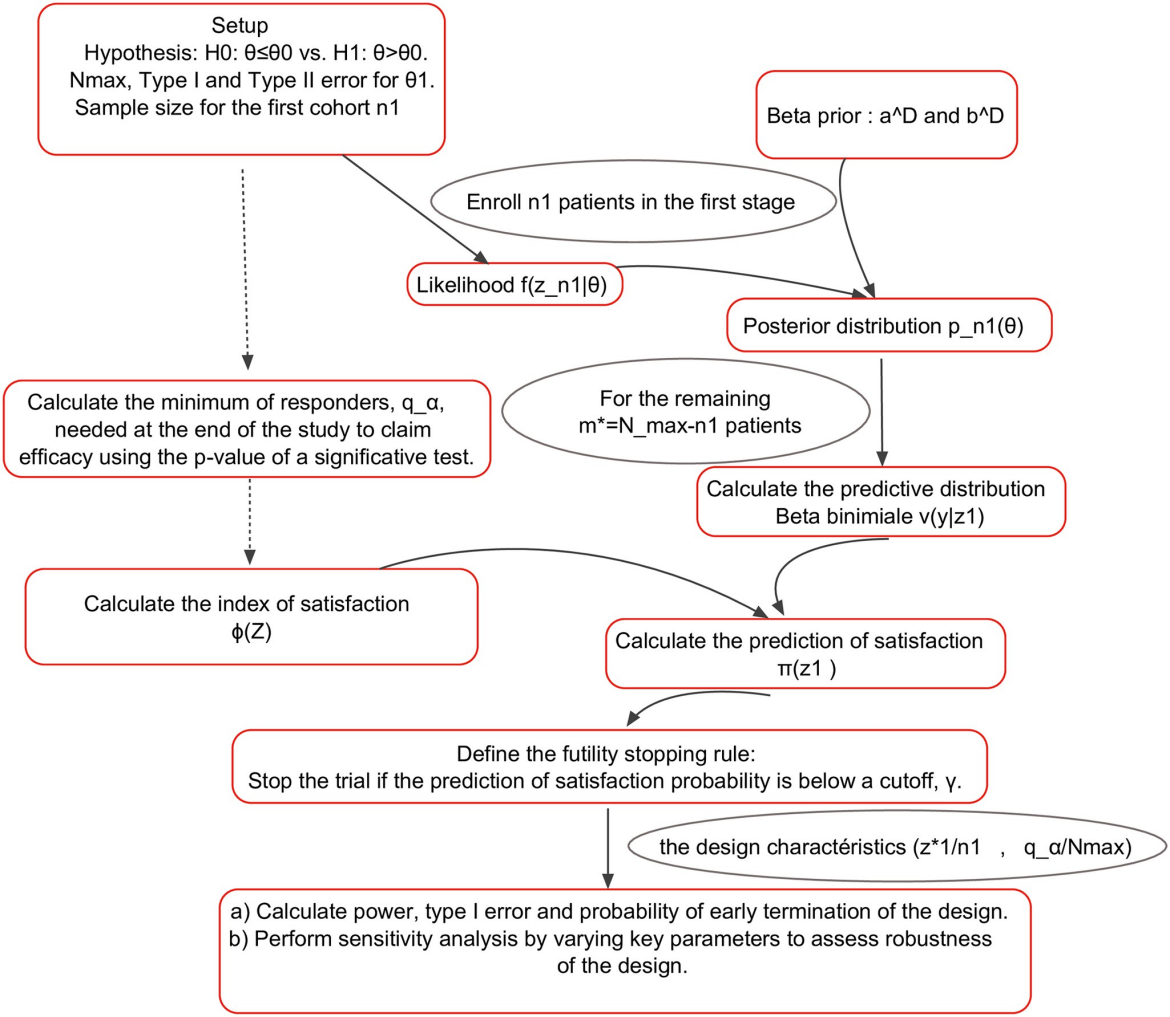
Flow chart of the PS-design for futility interim analysis.

The prediction of satisfaction is a simple but powerful concept to construct a phase II clinical design. Fig 1 summarizes the algorithm of the concept above.

### 2.2 Notations

The statistical model used in this method based on the unknown success probability parameter *θ* ∈ [0, 1]. Let denotes by *X* to the binary outcomes variable, such that:

$$\begin{array}{c} x_{i}=\{\begin{array}{cc} 1 & \text{if}\;\text{the}\;\text{treatment}\;\text{is}\;\text{a}\;\text{successful}\;\text{in}\;\text{a}\;\text{patient}\\ 0 & \text{otherwise} \end{array} \end{array}$$

The random “total number of success” acquired at the end of the trial out of *n* patients is denoted by $Z_{n}=\sum_{i=1}^{n}X_{i}$, with the binomial sampling distribution *B*(*n*, *θ*).

In a Bayesian conjugate assessment, we derive the posterior distribution by inserting the Beta prior for *θ* ∼ *p*(*θ*) = *Beta*(*θ*; *a*, *b*), such that:

$$\begin{array}{c} p_{n}\left(\theta |Z_{n}=z_{n}\right)=Beta\left(\theta ;z_{n}+a,n-z_{n}+b\right); \end{array}$$

The Choice and the elicitation of the prior is explained in the following section.

Furthermore, the Bayesian procedure has the advantage of allowing us to analytically determine the predictive distribution for the number of responses within a future sample given the recently updated response rate, which is, as is well known, a beta-binomial [14].

Let *θ*_0_ indicates a fixed value that represents the success probability for the control or standard therapy according to past data. Therefore, we assume that the therapy is promising if its efficacy probability surpasses the goal value *θ*_0_.

### 2.3 Elicitation of the prior

The prior distribution of the unknown parameter reflects past knowledge about the efficacy of the new therapy, and we utilize it to obtain: (1) the posterior probability and (2) the prediction distribution of the number of successes.

For binary observations, knowledge regarding the experimental treatment’s response data aids in determining the Beta prior distribution, *p*(*θ*) = *Beta*(*θ*; *a*, *b*). While many experimental therapies are generally the first in a series of studies, some involve a mix of standard therapy and a novel medicine or a change of regular treatment [6]. Thus, using historical data to better shape prior distribution within the Bayesian context should be beneficial.

The mean response rate of the beta distribution is $\bar{\theta }=\frac{a}{a+b}$. When the (*a* + *b*) grows, the confidence in prior information becomes stronger and will most likely influence the conclusions. So, the investigator can use this response rate estimate, which is the mean of the prior distribution of *θ* and an expression of his degree of confidence in $\bar{\theta }$ via a specific coefficient of variation of $\bar{\theta }$, $CV(\bar{\theta })$. A value of $CV(\bar{\theta })=10\%$ would indicate a high confidence that $\theta =\bar{\theta }$, $CV(\bar{\theta })=25\%$ moderate and $CV(\bar{\theta })=50\%$ low confidence etc…

Let $CV(\bar{\theta })=V.100\%$, for the Beta distribution’s hyper-parameters, we get:

$$\begin{array}{c} a^{D}=\left[\frac{1-\bar{\theta }}{V^{2}}\right]-\bar{\theta }\;\;\;and\;\;\;b^{D}=a^{D}\left[\frac{1}{\bar{\theta }}-1\right], \end{array}$$

where D indicates hyper-parameters of the design prior.

In the sequential use of Bayes theorem, the posterior from the first stage of the research simply turns into the prior for the second, and the ultimate posterior distribution follows in the same way [10].

### 2.4 Prediction of satisfaction in a binomial sequential setting

Designing a trial using the PS approach: Search the maximum sample size (*N*_*max*_), the cohort size of the current stage (*n*_*k*_), the minimum outcome in the current stage ($z_{k}^{*}$) and the maximum outcome in *N*_*max*_ (*q*_*α*_) that the treatment is considered efficient by the end of the trial such that the constraints of type I, type II and *γ* are satisfied as follows:

The sample size is assigned at each stage, with *n*_*k*_ representing the sample size of patients in the *k*^*th*^ interim analysis and $N_{max}=\sum_{k=1}^{K}n_{k}$ signifying the overall sample size. Generally, a design that has type I error and type II error satisfying the constraints will be selected.

In the interim look, by considering $z_{n_{k}}$, the number of responders in the current cohort (*n*_*k*_ patients), we should have $z_{N_{max}}=z_{n_{k}}+y$ responders if the trial goes to its term (to simplify it will be denoted by *z*). Then, the predictive density function of the number of responders among the remaining m* = *N*_*max*_ − *n*_*k*_ patients given the recently updated response rate, $y|z_{n_{k}}$, may be computed analytically by:

$$\begin{array}{c} \nu (y|z_{n_{k}};n_{k})=\frac{C_{m^{*}}^{y}\beta (y+z_{n_{k}}+a,N_{max}-(y+z_{n_{k}})+b)}{\beta (z_{n_{k}}+a,n_{k}-z_{n_{k}}+b)} \end{array}$$

for *y* = 0, …, m*. For a test of level *α*, where the null and alternative hypothesis are:

$$\begin{array}{c} H_{0}:\theta \leq \theta_{0}\;\;\;vs\;\;\;H_{1}:\theta >\theta_{0} \end{array}$$ (1)

where *θ*_0_ is the target value response rate of the desirable level of the treatment efficacy. Of course, rejecting “*H*_0_” means “accepting treatment efficacy”. In sequential trials, it is more interesting to consider at what level the final results will be in order to get a significant test. Then, the test *p*-value at the planned conclusion is defined by *p*(*z*) = inf{*ϵ*; *z* ∈ *R*^(*ϵ*)^}, where *R*^(*ϵ*)^ is the critical region. It has used to calculate the satisfaction of the investigator denoted by *ϕ*(*z*), and calculated by [12], as:

$$\begin{array}{c} \phi (z)=\{\begin{array}{ccc} 0 & \text{if} & z\notin R^{\left(\alpha \right)}\\ 1-p(z) & \text{if} & z\in R^{\left(\alpha \right)} \end{array} \end{array}$$

so the index of satisfaction for the binomial model will be:

$$\begin{array}{c} \phi \left(z\right)=B\left(z,N_{max},\theta_{0}\right)1_{\left\{z\geq q_{\alpha }\right\}} \end{array}$$

Where *B*(.) is the cumulative distribution function of the *binomial* model, and the rejection region is *R*^(*α*)^ = {*z* ≥ *q*_*α*_} with *q*_*α*_ = *inf*{*u*; *Pr*(*Z* ≥ *u*|*θ*_0_) ≤ *α*}.

Given economic/ethical restrictions, it is frequently unnecessary to continue a clinical study after an intermediate analysis if we can reliably predict that it will result in a significant conclusion. However, based on the intermediate analysis result $z_{n_{k}}$, it is possible to predict what the satisfaction will be at the conclusion of the experiment. By the stochastic curtailment methods, it is more interesting to forecast the degree of contentment by averaging the index of satisfaction with regard to the predictive probability over the entire space conditioned by the first stage result [10, 13]. Finally, for each interim analysis the prediction of satisfaction is updated by:

$$\begin{array}{ccc} \pi (z_{n_{k}}) & = & \sum_{y={\left(q_{\alpha }-z_{n_{k}}\right)}^{+}}^{m^{*}}(\phi (z_{n_{k}}+y))\nu (y|z_{n_{k}};n_{k})\\ & = & \sum_{y={\left(q_{\alpha }-z_{n_{k}}\right)}^{+}}^{m^{*}}(\sum_{t=0}^{y-1}C_{m^{*}}^{t}\;\theta_{0}^{t}{\left(1-\theta_{0}\right)}^{m^{*}-t})\frac{C_{m^{*}}^{y}\beta (y+z_{n_{k}}+a,N_{max}-(y+z_{n_{k}})+b)}{\beta (z_{n_{k}}+a,n_{k}-z_{n_{k}}+b)} \end{array}$$ (2)

This procedure can be used as a stopping rule for interim monitoring. In this case, the decision to stop the trial is based on the prediction of satisfaction exceeding a given threshold. However, we declare that the therapy is effective (or promising) if the prediction of satisfaction (given $z_{n_{k}}$ in the current stage) is higher than a threshold *γ*, (*γ* ∈ [0.5; 1]).

Note that $\pi \left(z_{n_{k}}\right)$ replaces the power of the test in the theory of the index of satisfaction recommended by Djeridi and Merabet [10]. The investigator is responsible for determining the threshold of the prediction of satisfaction in each intermediate analysis such that the pursuit of the experiment is discouraged.

For example, we define the design parameters as follows: $\theta_{0}=0.3,\;\bar{\theta }=0.5,\;CV=10\%,\;\alpha =0.1$, and 1 − *β* = 0.8 for a maximum sample size of *N*_*max*_ = 32 the frequentist one, and the design maximum sample size *N*_*max*_ = 27, for two scenarios (a) when the prior is informative (*p*(*θ*) = *Beta*(*a*^*D*^, *b*^*D*^)) (b) if the prior is non-informative (*p*(*θ*) = *Beta*(1, 1)). In this case, we plan an interim analysis after enrolling 10 patients; that is *K* = 1 and *n*_1_ = 10. Assume that the prediction of satisfaction (PS) threshold for inefficacy stopping is *γ* = 0.5. Table 1 displays the prediction of satisfaction for success numbers at the first interim look *z*_1_ = 0, …, 10. If *z*_1_ ≤ 2 the prediction of satisfaction *π*(*z*_1_) is smaller than 0.5 for the scenario (a) and if it is 3 or less for the scenario (b) (which is a consequence of the lack of information about *θ* in scenario (b)), and the study will be terminated due to ineffectiveness. On the other hand, continuing the experiment to the final analysis, the sample size was chosen by comparing the overall frequentist operating characteristics **section 2.5**.

**Table 1:** Prediction of satisfaction (PS) at the first interim look when *θ*_0_ = 0.3, *θ*_1_ = 0.5, *α* = 0.1, (1 − *β*) = 0.8 and *n*_1_ = 10 for two scenarios (a) *p*(*θ*) = *beta*(*a*^*D*^, *b*^*D*^) for $\bar{\theta }=0.5$ and *CV* = 10% and (b) *p*(*θ*) = *beta*(1, 1).

*x*_*i*_	*PS*(*p*(*θ*) = *Beta*(*a*^*D*^, *b*^*D*^))		*PS*(*p*(*θ*) = *Beta*(1, 1))	
	*N*_*max*_ = 27	*N*_*max*_ = 32	*N*_*max*_ = 27	*N*_*max*_ = 32
0	0.2119555	0.2796612	0.0014813	0.0018213
1	0.2325253	0.3060416	0.01051750	0.01324671
2	0.4143679	0.4814358	0.07231145	0.08043638
3	0.6118921	0.6567134	0.25504505	0.26315640
4	0.7721459	0.7952960	0.53935945	0.53897687
5	0.8670828	0.8808774	0.78521694	0.78308480
6	0.9083718	0.9224529	0.91450292	0.91823979
7	0.9239130	0.9399516	0.96685071	0.97227361
8	0.9319955	0.9484901	0.98841632	0.99160509
9	0.9385388	0.9484901	0.99694340	0.99807666
10	0.9445295	0.9598243	0.99954337	0.99975626

#### 2.4.1 Set up a stopping rule

As a stopping rule, predicting satisfaction is a useful tool. When it’s low, it can be used to end attempts prematurely due to futility. By setting a threshold *γ* (*γ* ∈ [0.5; 1]) for the prediction satisfaction to indicate an unfavorable probability of success, the number of responders $z_{n_{k}}^{*}$, for the stopping boundary at the *k*^*th*^ stage is the smallest number of success such that:

$$\pi (z_{n_{k}}^{*})\geq \gamma$$

is satisfied.

For example, for two stages trials, we establish the stage design that meets the error probability criteria and minimizes the maximum sample size to discover the minimax design or minimizes the predicted sample size to get the optimal design as the following:

For each value of total sample size *N*_*max*_ and each value of *n*_1_ in the range (10 : *N*_*max*_ − 10), we determine the smallest cohort size $n_{1}^{*}$ and $z_{1}^{*}=min\{z_{1}\in 0,\ldots ,n_{1}^{*}:\pi \left(z_{1}^{*}\right)\geq \gamma \}$ and verify the constraints of type I and type II errors. Of course, the maximum sample size *N*_*max*_ and the boundary level *q*_*α*_ required for the second stage drastically affect the values of $n_{1}^{*}$ and $z_{1}^{*}$. As a result, if the number of observed responders is less than or equal to the threshold $z_{1}^{*}$, we terminate the study for treatment ineffectiveness.

Analogously, if we agree to continue the study, we will get the (*K* − 1)^*th*^ observation $z_{K-1}^{*}$ in the final interim analysis. Therefore, we will need to plan a sample *y* with a cohort size of $\left(N_{max}-\sum_{i=1}^{K-1}n_{i}\right)$. Thus, we repeat the method to show an unfavorable chance of success and the number of responders $\left(z_{k}^{*}\right)$ for the stopping boundary at the *k*^*th*^ stage.

Another function is to stop trying validity if the prediction of satisfaction is very high. However, this is less feasible because of the early phase II study’s limited sample size [10].

### 2.5 Remarks about maximal sample size determination

The advantage of sequential methods results in saving sample size, time and cost when compared with standard static sample procedures. We determine the initial sample size *N* on the basis of the frequentist one stage which is

$$\begin{array}{c} \tilde{\theta }\left(1-\tilde{\theta }\right)\left[\frac{u_{1-\alpha }+u_{1-\beta }}{\theta_{1}-\theta_{0}}\right] \end{array}$$ (3)

where *u*_*ρ*_ is the standardized normal variate exceeded with probability *ρ* and $\tilde{\theta }=\frac{\left(\theta_{1}-\theta_{0}\right)}{2}$.

As there may be some uncertainty in the design prior during the planning phase, the preliminary size of the sample should be re-evaluated by utilizing interim data while there is one method to modify the maximal sample size *N*_*max*_, which involves revising it during interim evaluations. The requirement of such a large sample size will be prompted by ethical considerations as well as resource constraints. The search over *N*_*max*_ began with a value of (3). We verified below this beginning point to confirm that we had identified the least possible sample size *N*_*max*_ for which a non-trivial ($n_{i}\geq 0,\;i=\bar{1,k}$) multi-stage design met the error probability constraints. The enumeration algorithm looked upwards from this highest value of *N*_*max*_ until the optimum was clearly identified.

Table 2 display the needed sample size *N*_*max*_ for various criteria and values of *θ*_0_, $\bar{\theta }$, *CV*, *α* and (1 − *β*). We recognize that *CV* = 15% refers to around 0.20 for the 95% credible interval range. We can observe that the larger *α* and (1 − *β*) are in contrast to the other factors, the larger the sample size is. On the other hand, the choice of $\bar{\theta }$ has some influence on the sample size, and *CV* has a substantial effect if $\bar{\theta }$ is near to *θ*_0_. For all the cases, the PS design reduces the sample size as that obtained from the frequentist approach (the reduction is almost from 10% to 50%) that corresponds to the hypothesis testing framework (1), for the type I error *α* and a power 1 − *β*, if $\theta_{1}=\bar{\theta }$. Table 2 shows the frequentist findings in brackets.

**Table 2:** Sample size *N*_*max*_ for different criteria and values of *θ*_0_, *θ*_1_, *CV*, *α*, and 1 − *β* for (*p*(*θ*) = *Beta*(*a*^*D*^, *b*^*D*^)).

		$a^{D}=[\frac{1-\bar{\theta }}{V^{2}}]-\bar{\theta }\;and\;b^{D}=a^{D}[\frac{1}{\bar{\theta }}-1]$											
		$\bar{\theta }=\theta_{1}$											
		*θ*_0_ + 0.10				*θ*_0_ + 0.15				*θ*_0_ + 0.20			
		*CV*				*CV*				*CV*			
		15%		1.5%		15%		1.5%		15%		1.5%	
		*α*		*α*		*α*		*α*		*α*		*α*	
*θ*_0_	(1 − *β*)	0.2	0.1	0.2	0.1	0.2	0.1	0.2	0.1	0.2	0.1	0.2	0.1
0.2	0.7	29	59	25	54	14	23	14	23	11	14	7	12
				[48]	[72]			[26]	[38]			[17]	[22]
	0.8	44	84	43	72	25	38	30	38	14	25	7	18
				[70]	[98]			[34]	[47]			[22]	[30]
	0.9	76	122	75	122	39	55	39	55	25	35	20	33
				[102]	[135]			[48]	[68]			[30]	[42]
0.3	0.7	30	65	35	67	15	32	16	35	9	15	9	19
				[57]	[88]			[27]	[41]			[18]	[24]
	0.8	54	98	59	91	26	46	26	43	10	27	9	27
				[76]	[115]			[36]	[58]			[24]	[32]
	0.9	103	140	100	135	45	70	45	65	25	38	23	39
				[119]	[162]			[57]	[79]			[33]	[44]
0.4	0.7	37	77	36	76	16	37	16	37	17	17	10	17
				[62]	[97]			[26]	[45]			[16]	[29]
	0.8	60	112	60	100	28	50	27	51	17	27	16	28
				[83]	[125]			[41]	[58]			[25]	[34]
	0.9	110	162	106	155	54	71	52	67	27	40	27	40
				[126]	[179]			[55]	[83]			[34]	[49]
0.5	0.7	41	76	33	71	15	36	15	36	11	19	8	18
				[60]	[94]			[28]	[44]			[17]	[26]
	0.8	66	108	61	99	29	48	29	49	15	29	10	29
				[83]	[126]			[41]	[57]			[24]	[33]
	0.9	108	168	101	155	47	69	44	69	29	40	27	40
				[125]	[179]			[58]	[79]			[32]	[44]
0.6	0.7	42	69	37	69	17	28	18	28	9	10	9	9
				[57]	[92]			[27]	[42]			[16]	[24]
	0.8	62	96	64	93	26	40	32	44	17	25	9	25
				[76]	[119]			[35]	[53]			[22]	[30]
	0.9	100	146	117	160	44	60	59	71	23	34	26	34
				[115]	[162]			[52]	[73]			[30]	[42]
0.7	0.7	28	53	15	51	11	16	11	17	16	19	14	14
				[48]	[76]			[21]	[33]			[14]	[21]
	0.8	44	74	43	74	20	26	15	31	17	20	14	14
				[70]	[102]			[29]	[45]			[18]	[25]
	0.9	78	117	72	115	32	37	32	42	18	26	14	22
				[98]	[135]			[44]	[57]			[21]	[29]

The sample sizes in brackets were obtained using the frequentist technique for a binomial distribution.

### 2.6 Modelling considerations

Under the prediction of satisfaction framework, there are two approaches to Bayesian interim analysis described in this paper. The first one focuses on decision analysis that considers “the cost of decision errors and the prediction of satisfaction of the given outcomes in deciding whether to stop early”. In this approach, the design is specified by: the maximum sample size (*N*_*max*_), the cohort size of the current stage (*n*_*k*_), the minimum outcome in the current stage ($z_{k}^{*}$) and the maximum outcome in *N*_*max*_ (*q*_*α*_) that the treatment is considered efficient by the end of the trial such that the constraints of type I, type II and *γ* are satisfied (this approach is used to find the minimax design in the previous section). Another approach is to calculate the prediction of satisfaction for any maximum sample size, for any size of interim cohort to find the design’s plan. This design requires no appeal to use the power for its evaluation. However, in both cases, once the rule is defined, we have to determine the design frequentist operation characteristics (type I and type II errors, the probability of early termination and the expected sample size).

Because the stopping rule characterizes the study design, we have to provide the stopping boundary ($z_{k}^{*}/n_{k},\;q_{\alpha }/N_{max}$). The frequentist operating characteristics functions are, then, given by:

1- Let *b*(.) and *B*(.) be the probability mass function and the cumulative distribution function of the binomial distribution, respectively. The rejection probability after the first stage is:

$$\begin{array}{c} Pr\left(rejecting\;H_{0}|\theta \right)=B\left(z_{1}^{*},n_{1},\theta \right) \end{array}$$

Hence, in this design, the probability of early termination (PET) after the first stage is:

$$\begin{array}{c} PET\left(\theta \right)=B\left(z_{1}^{*},n_{1},\theta \right) \end{array}$$

Furthermore, the expected sample size is:

$$\begin{array}{c} E\left(N|\theta \right)=n_{1}\times PET\left(\theta \right)+N_{max}\times \left(1-PET\left(\theta \right)\right) \end{array}$$

2- For a three stage design, the rejection probability becomes:

$$\begin{array}{c} Pr\left(rejecting\;H_{0}|\theta \right)=1-B\left(z_{1}^{*},n_{1},\theta \right)-\sum_{i=z_{1}^{*}+1}^{min(n_{1},q_{\alpha }-1)}b\left(i,n_{1},\theta \right)B\left(q_{\alpha }-1-i,N_{max}-n_{1},\theta \right) \end{array}$$

the probability of early termination (PET) is then:

$$\begin{array}{c} PET\left(\theta \right)=B\left(z_{1}^{*},n_{1},\theta \right)+\sum_{i=z_{1}^{*}+1}^{min(n_{1},q_{\alpha })}b\left(i,n_{1},\theta \right)B\left(q_{\alpha }-1-i,N_{max}-n_{1},\theta \right) \end{array}$$

3- For a *k*−stage design, (*k* > 3), the rejection probability becomes:

$$\begin{array}{c} Pr\left(rejecting\;H_{0}|\theta \right)=1-B\left(z_{1}^{*},n_{1},\theta \right)-\sum_{i=z_{1}^{*}+1}^{min(n_{1},q_{\alpha }-1)}b\left(i,n_{1},\theta \right)B\left(q_{\alpha }-i,N_{max}-n_{1},\theta \right)\\ -\ldots -\sum_{i=z_{k}^{*}+1}^{min(n_{k-1},q_{\alpha }-1)}\left[\prod_{l=1}^{k-1}b\left(t_{l},n_{l},\theta ;t_{l}>z_{l}^{*},\sum t_{l}=i\right)\right]\\ \times B(q_{\alpha }-1-i,N_{max}-\sum_{j=1}^{k}n_{j},\theta ) \end{array}$$

the probability of early termination (PET) is then:

$$\begin{array}{ccc} PET(\theta ) & = & B\left(z_{1}^{*},n_{1},\theta \right)+\sum_{i=z_{1}^{*}+1}^{min(n_{1},q_{\alpha })}b\left(i,n_{1},\theta \right)B\left(q_{\alpha }-1-i,N_{max}-n_{1},\theta \right)+\ldots \\ & - & \sum_{i=z_{k}^{*}+1}^{min(n_{k-1},q_{\alpha }-1)}\left[\prod_{l=1}^{k-1}b\left(t_{l},n_{l},\theta ;t_{l}>z_{l}^{*},\sum t_{l}=i\right)\right]B\left(q_{\alpha }-1-i,N_{max}-\sum_{j=1}^{k}n_{j},\theta \right) \end{array}$$

4- Finally,

$$Type\;I\;error=Pr(rejecting\;H_{0}|\theta_{0})\;\text{and}\;power=Pr(rejecting\;H_{0}|\theta_{1})$$

## 3 Numerical evaluation

The benchmark for studying the proposed design is type I and type II errors and PET. The most well-known frequentist-based multi-stage designs for phase II clinical trials try to control such errors at pre-specified levels (see [9, 15]). The small variation in operating characteristics between admissible plans indicates the robustness of these plans to a slight variation in stage specific sample sizes actually attained [16]. As a result, it may be interesting to demonstrate how the suggested design performs in terms of these two types of error probabilities with the probability of early termination.

### 3.1 Sensitivity analysis of prediction of satisfaction plans

Conducting sensitivity analysis offers a chance to assess the durability of the plan and optimize it by modifying crucial variables. Four performance-related criteria are investigated: (i) *γ*, the threshold of the prediction of satisfaction, (ii) *α*, type I error rate, (iii) *N*_*max*_, the maximum sample size, (iv) The hyper-parameters of the prior distribution. When all other factors are held constant, the influence of each parameter is studied. We investigate three cases: two, three and multi-stage. The design plans are derived numerically by parallel computing utilizing a self-written algorithm in R.

### 3.2 Results

We have considered *θ*_0_ = 30% as a suitable objective level of treatment effectiveness probability, *θ*_1_ = 50% (the minimum favourable response rate). The degree of significance *α* is taken as 5% and 10% with a type II error of 20%. The predefined sample size is *N*_*max*_ = 50 and assume the uniform prior distribution (*p*(*θ*) = *Beta*(1, 1)). If the prediction of satisfaction is more than 0.5, the therapy is regarded promising. Thus, with a total of 50 patients and to assert efficacy, we will require at least 20 responses (i.e., *q*_*α*_ = 20) by the index of satisfaction for *α* = 0.05 and at least 19 responders (i.e., *q*_*α*_ = 19), if *α* = 0.10.

Table 3 presented two-stage, three-stage and 5 multi-stage PS plans for comparison that could be.

**Table 3:** Prediction of satisfaction at the first interim look when *θ*_0_ = 0.3, *θ*_1_ = 0.5, for two scenarios (a)*α* = 0.05 and (b) *α* = 0.10, where (1 − *β*) = 0.8 and *n*_1_ = 10.

Example n°	*α* = 0.05			*α* = 0.10		
*K* (number of stages)	Two-stages	Three-stages	Multi-stages	Two-stages	Three-stages	Multi-stages
*n*_*k*_(Sample size at each stage)	25, 50	15, 15, 20	10, 10, 10, 10, 10	25, 50	15, 15, 20	10, 10, 10, 10, 10
$\sum_{j=1}^{k}\;n_{j}$	25, 50	15, 30, 50	10, 20, 30, 40, 50	25, 50	15, 30, 50	10, 20, 30, 40, 50
$z_{k}^{*}$ stopping boundary	11, 20	7, 13, 20	5, 9, 13, 17, 20	10, 19	6, 12, 19	5, 8, 12, 16, 19
*PS* at stage *k*	0.74	0.69, 0.73	0.72, 0.66, 0.64, 0.79	0.64	0.56, 0.66	0.72, 0.57, 0.66, 0.70
Performance						
TypeI error	0.044	0.048	0.03	0.098	0.08	0.06
Power	0.79	0.82	0.87	0.89	0.92	0.92
PET	0.90	0.89	0.89	0.81	0.90	0.92
*E*(*N*|*θ*_0_)	34.02	44.97	43.12	33.11	39.18	43.30
*E*(*N*|*θ*_1_)	27.12	25.33	19	26.15	19.79	20.35

The risk of type I errors decreases as the number of intermediate looks increases. This is due to the use of the p-value which reserves the level of the overall significance of the repeated testing hypothesis with accumulating data [17]. Conversely, type II errors slightly decrease. Furthermore, we realized that when the number of interim observations grows, PET increases somewhat and the predicted sample size, *E*(*N*/*θ*_1_), drops. On the other hand, the expected sample size, when *θ* = *θ*_1_, is smaller than the expected sample size when *θ* = *θ*_0_, because the prior confidence is specified in a good treatment.

The prediction of satisfaction design has generally a larger boundary, $z_{k}^{*}$, when type I error rate is smaller, which effects type II error probability that becomes smaller. So, for controlling type II error probability, a larger *n*_*k*_ is needed. Thus, although $z_{k}^{*}$ (and therefore PET) under the null, is higher, the PS design still has a larger expected sample size for a small type I error rate, but still less than for one stage design.

### 3.3 Sensitivity analysis for the two-stage strategy

**Fig 2:**
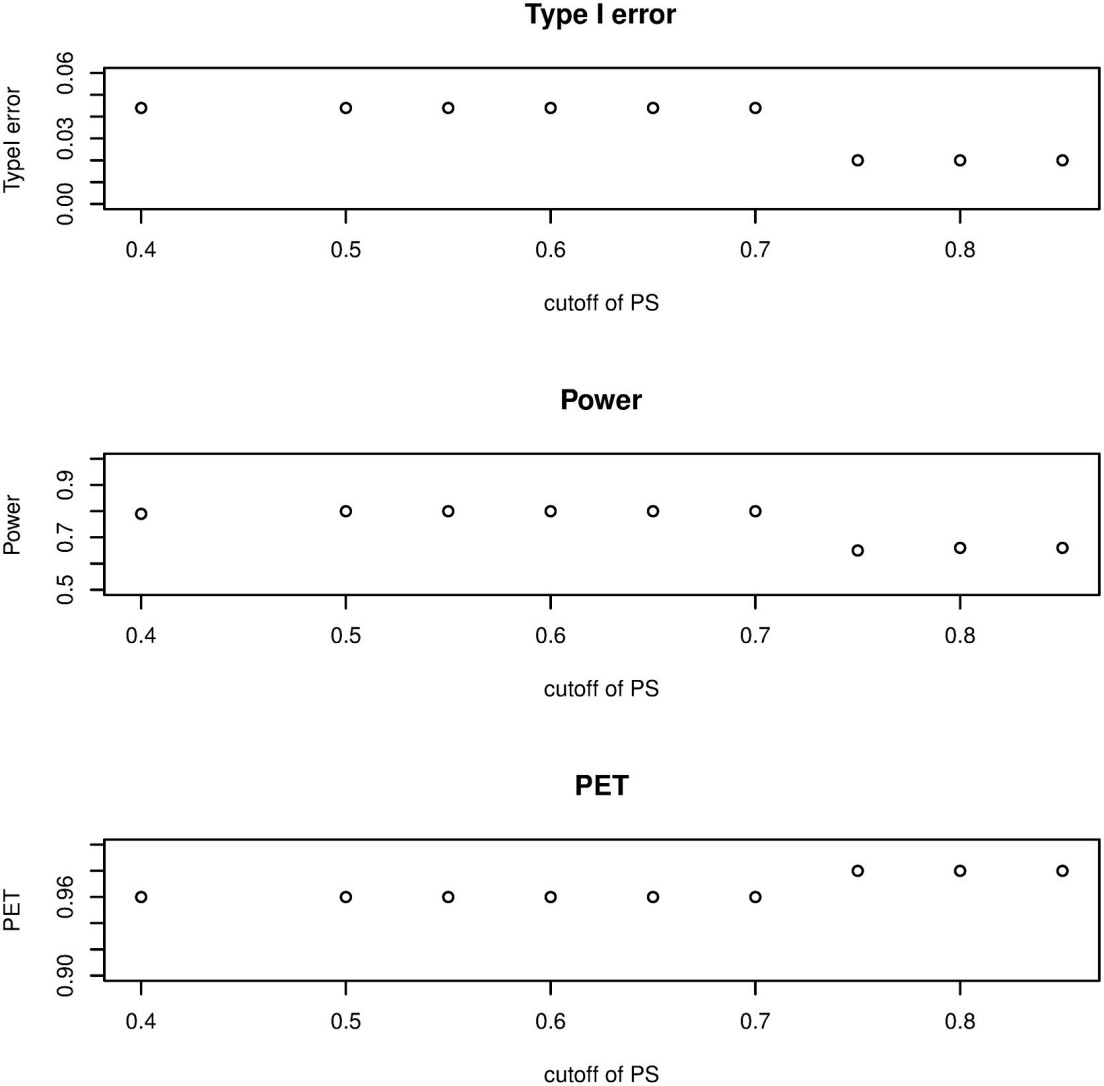
Sensitivity analysis in two-stage plan by altering the threshold for predicting satisfaction.

**Fig 3:**
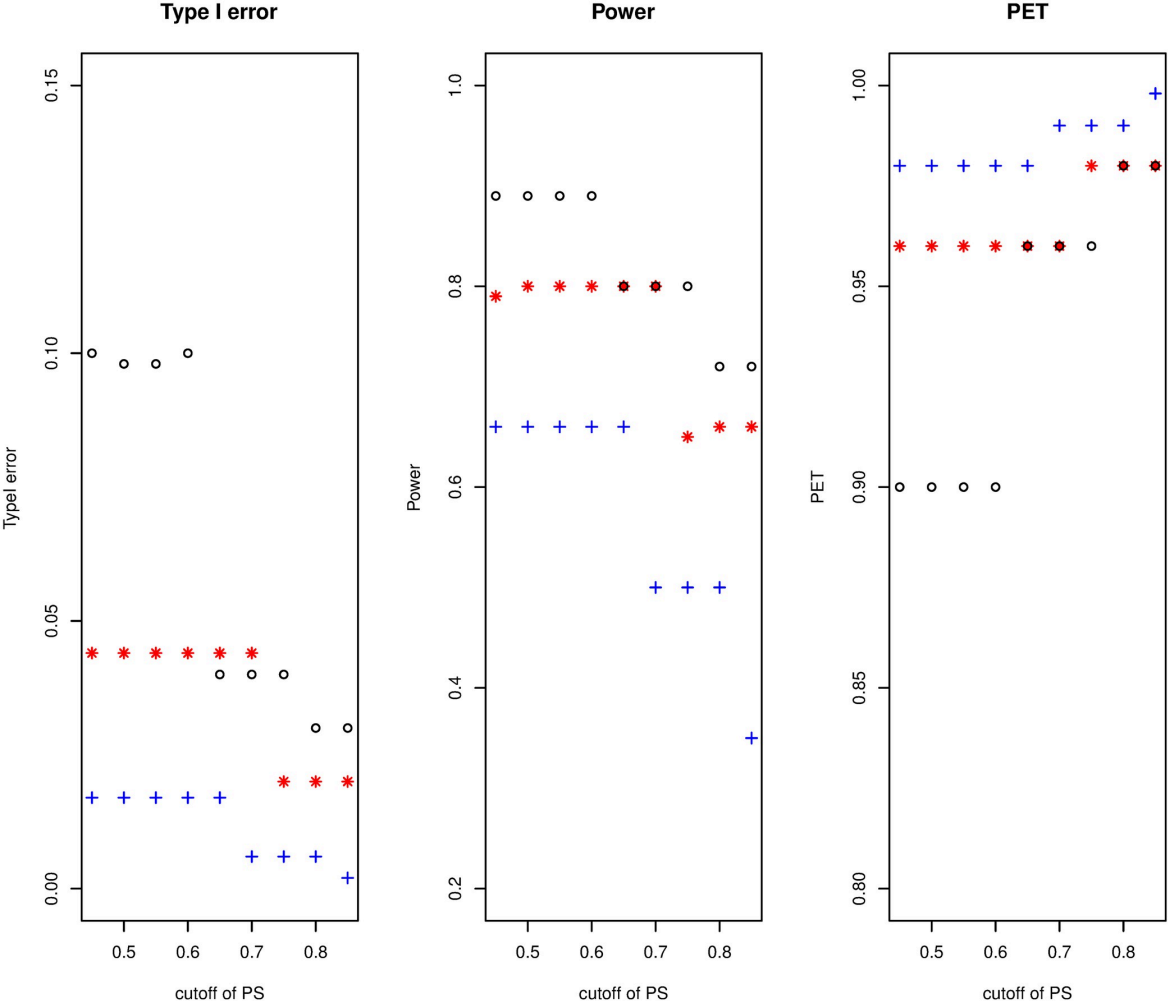
Sensitivity investigation of the combined effect of both threshold of the prediction of satisfaction and *α* in the two-stage example.

**Fig 4:**
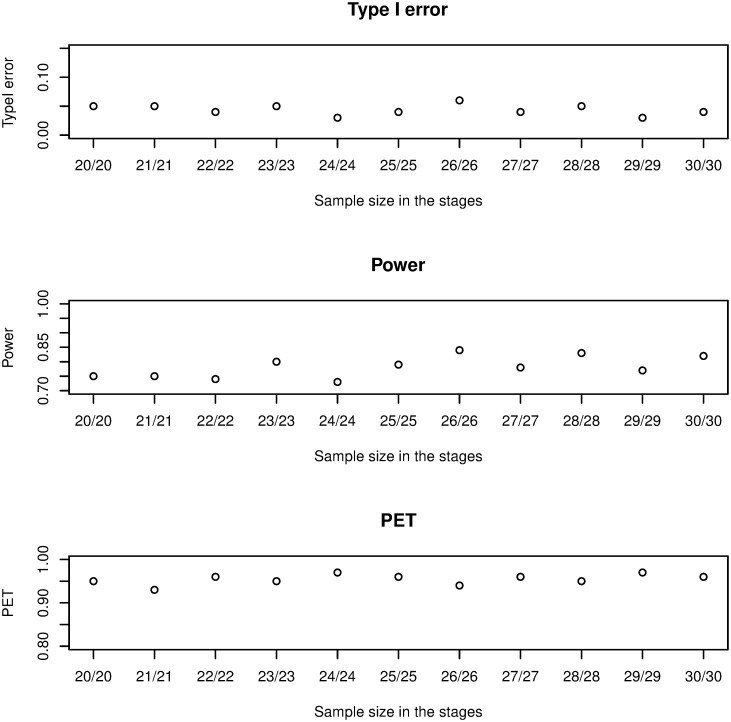
Sensitivity analysis by changing sample size in the two-stage plan.

**Fig 5:**
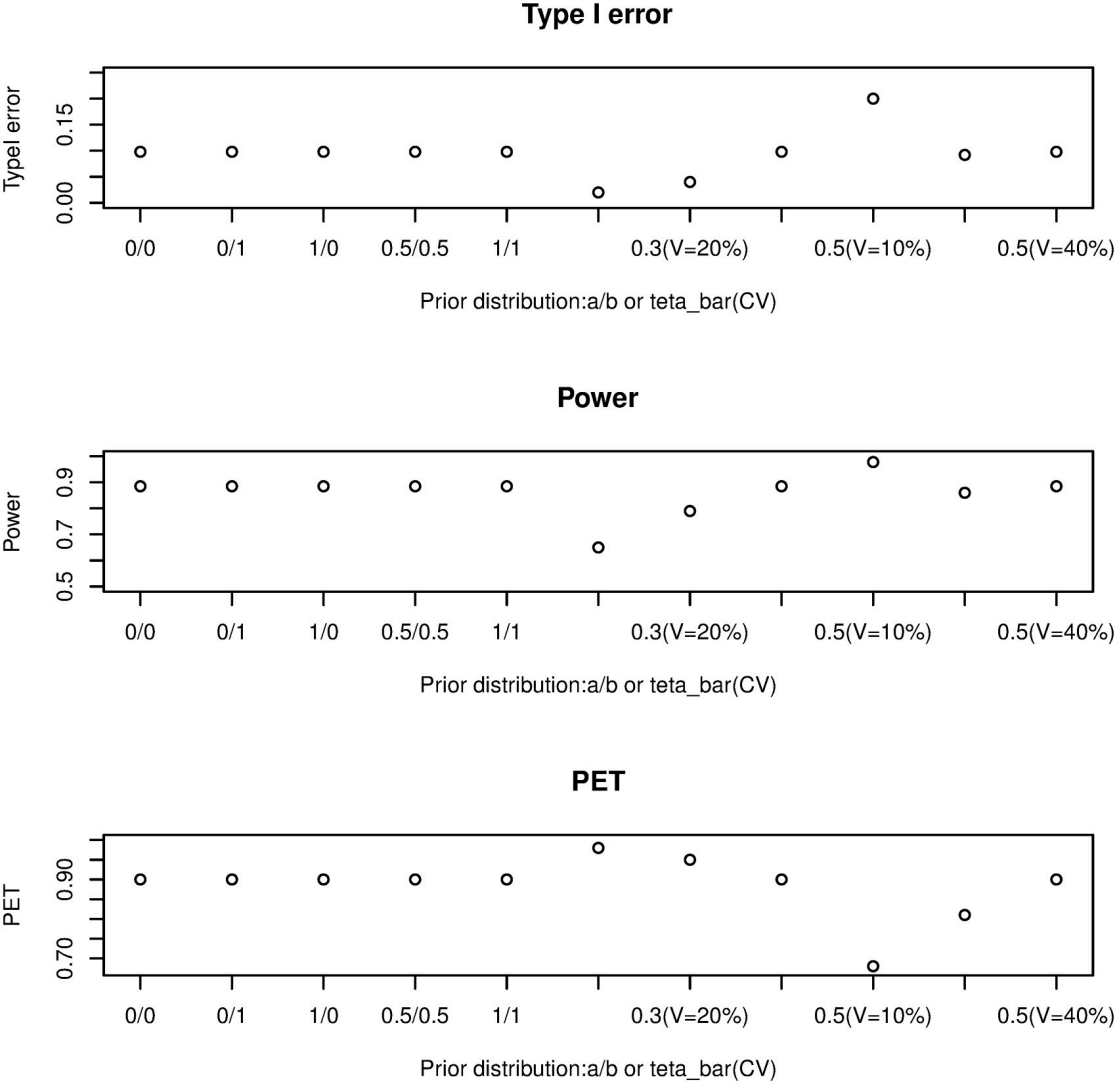
Sensitivity analysis by altering prior hyper-parameters in the two-stage plan.

To summarise, the study of the essential parameters to assess performance of the two-stage design are illustrated by:

(i) Threshold of the prediction of satisfaction: as it is shown in Fig 2, if *γ* increases from 0.40 to 0.85, type I error and power will decrease (from 0.05 down to 0.015 and from 0.85 down 0.41, respectively) but PET increases (from 0.96 to 0.98). The influence is significant on the type I error, mild on power, and minor on PET.
(ii) The join effect of *α* and *γ*: by considering (*α* = 0.01, 0.05, *and* 0.10) with the threshold of the PS is from 0.40 to 0.85, we investigate the combined impact. It is evident, in Fig 3, that PET increases as *γ* increases while type I error and power decrease. The difference between the values of the three metrics becomes negligible as *γ* increases, especially for PET. According to the findings, the join effect has a high influence on Type I error and PET and a middle effect on power.
(iii) Cohort size: a change in sample size has little effect on power or PET, but it reduces the type I error rate (Fig 4). The impact is middle on the three metrics and this is due to the discreteness of the model’s distributions.
(iv) Beta prior: the result has shown that the non-informative priors have little impact on the three metrics, because both parameters display the amount of responses and non-responses (Fig 5).

A comparable impact is produced by a prior determined by response rate at the null or alternative hypothesis with a large *CV* (example *CV* ≥ 0.4). In contrast, if *CV* is small (example *CV* ≤ 0.4), (*a* + *b*) significantly controls the conclusions. For example, when $\bar{\theta }=0.3$ with *CV* = 10%, *a* = 69.7 and *b* = 162.63, to claim effectiveness, it will require at least 19 responders (out of 50 patients). If there were 11 responders or less in the first stage, the experiment would be terminated early. As a consequence, the experiment might be ended in the first stage with *PET* = 98% and low power 65%. However, for a prior with a response rate of $\bar{\theta }=0.5$ and a *CV* of 10%, at least 8 responders are required to declare efficacy. In this case, *PET* = 68% with a power of 98% and a large type I error (0.32).

### 3.4 Detailed sensitivity analysis for multi-stage plan

The three and the multi-stage plans assume the uniform prior distribution. Each of these plans has slightly greater power and a slightly smaller significance level with a smaller *E*(*N*/*θ*_1_) than the two-stage plan due to the early termination opportunities they provide. The different stage cases show that as the number of interim analysis increases, the power increases and the type I error rate decreases. Moreover, due to the strict condition of using the p-value of the final significance conclusion, the stopping boundaries in the different plans are larger, so the probability of early termination is bigger than the other trial designs.

## 4 An illustrative example

The major goal of this research is to see how effectively a combination of nivolumab, ipilimumab, and nintidanib works in advanced non-small cell lung cancer (NSCLC) (NCT03377023 on clinicaltrials.gov).

A phase II single-arm trial is conducted for two cohorts: immunotherapy naïve and immunotherapy treated previously. The overall response rate (ORR) was our primary end objective, with success defined as a complete or partial response. Each cohort’s sample size was set at 40 patients. In each stage, the cohort includes 20 patients.

For the immunotherapy naïve cohort: consider a null hypothesis of 0.30 ORR and an alternative hypothesis of 0.50 ORR, with a one-sided type I error of 0.05 and a power of 0.8. As a consequence of analyzing 40 eligible patients, the final ORR was 42.5% (17/40). Partial responses were seen in 9 and 17 of the first 20 and 40 recruited patients, respectively (no complete response). To demonstrate the suggested technique, we give design parameters such as *θ*_0_, *α* = 0.05, *K* = 2, *n*_1_ = *n*_2_ = 20, *N*_*max*_ = 40. Assume the probability criterion for stopping futility is ≤ 0.5. After the first step, and with no knowledge on the unknown parameter *θ*, the flat prior *Beta*(1, 1) is optimal. Because the ORR in this interim investigation was 45% (9/20), the predicted level of satisfaction is 0.66 > 0.50. The experiment will not be terminated due to inefficacy, and the therapy merits further investigation in phase IIB or III studies. In this case, the operating characteristics were type I error = 0.05, power = 0.75 and *PET* = 95% (Table 4).

**Table 4:** Comparison of prediction of satisfaction to Bayesian predictive probability design.

method	$z_{n}^{*}$	*n*	*q*_*α*_	*N*_*max*_	$\pi (z_{n}^{*})$	type I error	power	PET
Immunotherapy naïve cohort: 30% *vs* 50% ORR								
PS design	9	20	17	40	0.67	0.05	0.75	0.95
PP design	6	20	16	40	0.08	0.06	0.85	0.61
immunotherapy treated previously cohort: 7% *vs* 20% ORR								
PS design	3	20	6	40	0.63	0.05	0.80	0.95
PP design	1	20	5	40	0.08	0.05	0.82	0.59

For the cohort of immunotherapy treated previously : let the null hypothesis be set as 0.07 ORR versus an alternative hypothesis about 0.20 ORR, by considering one sided type I error = 0.05 and a power of 0.8. The therapy is treated as effective if a total of responders are ≥ 6 (15%(6/40) of ORR), and the stopping rule is ≤ 2. The prediction of satisfaction is *π*(*x*_1_ = 3) = 0.63, the design has 5% type I error, 80% power and 95% of PET.

### 4.1 Comparison of the prediction of satisfaction with the predictive probability design

Lee and Liu in 2008, [8], suggested a Bayesian phase II design in which the futility criterion is based on an evaluation of the predicted probability that the trial would yield a conclusive result at the intended end of the research, given the observed data, at any interim analysis.

Consider in the current *n* patients, *z*_*n*_ responses are observed and let *Y* be the random variable reflecting the number of probable future *N*_*max*_ − *n* patients’ responses It is generally considered that the posterior predictive distribution of *Y* is

$$\begin{array}{c} \nu \left(y|z_{n},n\right)=beta-binom\left(y;N_{max}-n,a^{D}+z_{n},b^{D}+n-z_{n}\right),\;for\;\;y=0,1,\ldots ,\;\left(N_{max}-n\right). \end{array}$$

When the investigation is completed and the outcome *y* is obtained, the experimental therapy will be considered sufficiently promising if the criteria that follows is achieved:

$$\begin{array}{c} p_{n}\left(\theta >\theta_{0}|z_{n}+y,N_{max}\right)>\theta_{T}, \end{array}$$

where *θ*_*T*_ is a pre-specified probability threshold. Meanwhile, as *Y* has not yet been realized, the experiment should be carried out, and the posterior predictive distribution may be used to compute the probability of a positive conclusion for the maximum intended sample size, that is:

$$\begin{array}{c} PP=\sum_{y=0}^{N_{max}-n}\nu \left(y|z_{n},n\right)I_{\left\{p_{n}\left(\theta >\theta_{0}|z_{n}+y,N_{max}\right)>\theta_{T}\right\}}, \end{array}$$

where *I*_{.}_ refers to the indicator function. A low PP score suggests that the novel drug will most likely be ruled inefficient by the end of the trial.

Using the predictive probability design with the same flat prior and a threshold of 20% for the PP, the trial of immunotherapy naïve will stop for futility if $\left(z_{n}^{*}/n;q_{\alpha }/N_{max})=(6/20;16/40\right)$ patients observed. While for the trial of immunotherapy treated previously, the stopping boundaries are $\left(z_{n}^{*}/n;q_{\alpha }/N_{max})=(1/20;5/40\right)$. For the two trials, the prediction of satisfaction was 0.08 which would not meet the stopping rules. Moreover, the prediction of satisfaction has a higher PET and smaller power. This is due to the process of calibration of the probability thresholds, which assures a type I error rate of 0.05 and a power of 80% (Table 4). Therefore, as more patients are enrolled, more PET is obtained by using progressively stricter futility rules.

## 5 Discussion

Sequential analyses in clinical trials constitute an essential for maintaining overall quality standards, and such analyses may be critical if clinical trials are to be ethically accepted. The main drawback of sequential analyses is the raised risk of type I error because of the repeated tests. So the adoption of the index of satisfaction approach, [10], which uses the p-value of a significance result if the trial continues to its term, can resolve this problem. The prediction of satisfaction has been advocated as statistical evidence for early termination of phase II clinical trials [12].

In particular a smaller sample size does not necessary imply better design but the PS-design reduces the sample size as that obtained from the frequentist approach (the reduction is almost from 10% to 50%). Indeed, for a given prior distribution the sample size required by the PS-design may decline as *θ*_1_ increases. For example, when *θ*_1_ = *θ*_0_ + 10 for *θ*_0_ = 0.5, *CV* = 15%, *typeIerror* = 0.10, *and power* = 0.80, the sample size *N*_*max*_ = 108 but for *θ*_1_ = *θ*_0_ + 20 with the same other parameters it decreases to 29 (the case of rare promising treatment). Ideally, we would like to have *N*_*max*_ increases as *θ*_1_ increases.

Inference based on prediction of satisfaction combines the advantages of Bayesian and frequentist methods of inference by allowing for inference conditional on observed data, providing for incorporation of prior information, providing a logical compromise between the relative might of accumulated data and prior information and allowing for the assessment of significance levels and power in the sample space of future observations. In the sensitivity analysis section, we showed that in different phases, the PS-design has generally a larger boundary, $z_{k}^{*}$, when type I error rate is smaller, which affect type II error probability that becomes smaller. So a larger cohort size, *n*_*k*_, is needed to control type II error probability. Thus although $z_{k}^{*}$ and therefore the probability of early termination under the null, is higher, the PS-design still has a larger expected sample size for a small type I error rate, but still less than for one stage design.

We have summarized the perspective of this hybrid frequentist-Bayesian sequential design and discussed its implications. In addition, we evaluated many experimental situations to determine its simulated operational characteristics. To analyse this influence, we conducted replicated simulations to demonstrate how optimality is connected to the intricate interplay between type I error, the threshold *γ*, sample size, and prior information. Overall, the prediction of satisfaction design achieves a satisfactory sensitivity analysis if a significant number of responses are observed throughout the recruiting phase to allow the adaptive technique to work. Furthermore, even in experimental scenarios with no prior information, a high level of satisfaction is reached in favour of the most promising therapy. The feasibility of our concept was further demonstrated by redesigning an ongoing real lung cancer trial for immunotherapy.

From a practical standpoint, our design does not require intensive computation for sample size searching and there are few parameters to control. Furthermore, simple simulation is required to evaluate the operating characteristics at the design phase. Moreover, due to the strict condition of using the p-value of the final significance conclusion, the stopping boundaries in the different plans are larger, so the probability of early termination is bigger than the other trial designs like the PP-design as it was shown in the illustrative example. The authors are currently working on creating a user-friendly interface (R package) to apply the technique, which will be released in the near future.

In conclusion, we believe that the prediction of satisfaction design should be seen as an alternative among the usual adaptive designs for interim futility analysis in single arm phase II trials. These allow us to generally answer the experimenter questions about the stability of already observed results to future data. It can be used to advantage in the context of the sequential interim analysis. This approach appears to be of particular relevance in determining the sample size required to reach the desired conclusion.
